# The Impact of Meteorological Factors on Communicable Disease Incidence and Its Projection: A Systematic Review

**DOI:** 10.3390/ijerph182111117

**Published:** 2021-10-22

**Authors:** Mazni Baharom, Norfazilah Ahmad, Rozita Hod, Fadly Syah Arsad, Fredolin Tangang

**Affiliations:** 1 Department of Community Health, Faculty of Medicine, Universiti Kebangsaan Malaysia, Bandar Tun Razak, Kuala Lumpur 56000, Malaysia; mazni_baharom@yahoo.com (M.B.); rozita.hod@ppukm.ukm.edu.my (R.H.); fadlysyaharsad@gmail.com (F.S.A.); 2 Department of Earth Sciences and Environment, Faculty of Science and Technology, Universiti Kebangsaan Malaysia, Bangi 43600, Malaysia; tangang@ukm.edu.my

**Keywords:** climate change, global warming, meteorological factors, communicable disease projection, dengue, malaria, cholera, leptospirosis

## Abstract

Background: Climate change poses a real challenge and has contributed to causing the emergence and re-emergence of many communicable diseases of public health importance. Here, we reviewed scientific studies on the relationship between meteorological factors and the occurrence of dengue, malaria, cholera, and leptospirosis, and synthesized the key findings on communicable disease projection in the event of global warming. Method: This systematic review was conducted according to the Preferred Reporting Items for Systematic Reviews and Meta-Analyses (PRISMA) 2020 flow checklist. Four databases (Web of Science, Ovid MEDLINE, Scopus, EBSCOhost) were searched for articles published from 2005 to 2020. The eligible articles were evaluated using a modified scale of a checklist designed for assessing the quality of ecological studies. Results: A total of 38 studies were included in the review. Precipitation and temperature were most frequently associated with the selected climate-sensitive communicable diseases. A climate change scenario simulation projected that dengue, malaria, and cholera incidence would increase based on regional climate responses. Conclusion: Precipitation and temperature are important meteorological factors that influence the incidence of climate-sensitive communicable diseases. Future studies need to consider more determinants affecting precipitation and temperature fluctuations for better simulation and prediction of the incidence of climate-sensitive communicable diseases.

## 1. Introduction

In the last decades, the global burden of disease has shifted from communicable to non-communicable causes [1]. The coronavirus disease 2019 (COVID-19) pandemic on the other hand has demonstrated how communicable disease remains a significant threat to global health, particularly as the climate crisis continues to influence disease spread in a variety of ways. The evidence shows that the global surface temperature during the most recent decade (2011–2020) was 1.09 [0.95 to 1.20] °C higher relative to the pre-industrial period (1850–1900), which was driven by human activities [2]. Based on the Sixth Assessment Report of the Intergovernmental Panel on Climate Change, the global surface temperature will continue to rise, ranging from 1.5 to 4.4 °C in the twenty-first century. The global warming of 2 °C is likely to occur in the mid-term period (2041–2060) under the high greenhouse gases emissions scenario (Shared Socioeconomic Pathways, SSP3-7.0) [2]. A warming of 2 °C and more poses greater risk to human health [3], particularly on vulnerable subpopulations such as the elderly, low-income populations, and people with comorbidities [4,5].

Climate change poses a real challenge to public health and has contributed to causing the emergence and re-emergence of many communicable diseases of public health importance [5]. Climate change impacts communicable disease in many different ways through ecosystem changes or by disrupting disease control efforts. Among the communicable diseases, vector-borne and water-borne diseases are the two main categories predicted to be most affected [6]. Vector-borne diseases such as dengue [7,8] and malaria [9,10], water-borne disease such as cholera [11,12] and leptospirosis [13,14] were well known to be highly sensitive to climatic factors. An increase in global surface temperature and ocean temperature causes the sea level to rise. Rising sea levels will affect low-lying areas and cause seawater intrusion to coastal rivers and freshwater, and frequent flooding events [3]. Under these conditions, the transmission of water-borne disease such as cholera and leptospirosis was markedly increased. If stagnant water remains after a flood, the risk of vector-borne illnesses (such as malaria or dengue fever) increases [15]. Due to the increased global burden of dengue and leptospirosis [16,17], as well as increased mortality of malaria and cholera, particularly among children in developing countries [18], hence this review focuses on four highly climate-sensitive communicable diseases, namely dengue, malaria, cholera, and leptospirosis. Climate change has the greatest impact on developing countries, yet they are also the least able to cope with its consequences. Multiple factors contribute to their vulnerability, limiting their ability to prevent and adapt to the effects of climate change [19].

While the impacts of climate change on communicable diseases have been observed worldwide, the magnitude and types of effects vary based on the location of the country and its socioeconomic circumstances [3,5]. Risks from vector-borne and water-borne diseases are expected to surge with potential shifts in their geographic range due to changes in temperature and water imbalances [3]. Understanding this emerging field is essential for future mitigation, adaptation, and control measures in this issue. There are several gaps in the evidence base linking climate change to communicable disease [12], particularly on the role of meteorological factors and intra-annual variability as opposed to long-term climate change projections related to disease risk [5]. Assessing the projected communicable disease projection due to climate change would be an essential step for public health agencies to prepare for the climate change impacts. By examining the projection studies, besides enhancing the understanding of pathway linking the exposure to the health outcome, the key findings such as data needs for the modelling effort, type of global climate model (GCM) used, and mathematical model used for analysis can be obtained [20]. Understanding these findings are important for developing early warning systems and adaptation strategies to strengthen climate resilience in facing the impending impacts of 1.5/2.0 °C global warming. An effective early warning system will enhance outbreak preparedness and control, subsequently minimize the health and economic burdens of communicable diseases.

To guide future research and action to mitigate and adapt to the health impacts of climate change, particularly on communicable disease, a complete and thorough overview of the current research is needed. In the present study, we reviewed scientific studies on the relationship between meteorological factors and the occurrence of dengue, malaria, cholera, and leptospirosis, and synthesized the key findings on communicable disease projection in the event of global warming.

## 2. Materials and Methods

This systematic review is registered with PROSPERO (CRD42021239975) and is reported in accordance with the Preferred Reporting Items for Systematic Reviews and Meta-Analyses (PRISMA) 2020 statement [21].

### 2.1. Research Question Formulation

The review question was developed based on the PEO concept [22]. Generally, systematic reviews have been used to evaluate the efficacy of health interventions by critically evaluating and summarizing the findings of randomized controlled trials. Therefore, the PICO approach often has been used as guidance to develop the review question [23]. However, this systematic review is an aetiology/risk type of review, which aims to determine the association between particular exposures/risk factors and health outcomes. Thus, the PEO concept, which comprises population, exposure of interest (independent variable), and the outcome (dependent variable), was recommended [23]. Based on this concept, population refers to general population, exposure of interest is meteorological factors, and outcome is the occurrence of the selected communicable diseases, namely dengue, malaria, cholera, and leptospirosis. The PEO concept guided the formulation of the main review question: What is the impact of meteorological factors on communicable disease incidence (i.e., dengue, malaria, cholera, leptospirosis)? The second review question was: What is the communicable disease projection due to climate change?

### 2.2. Data Sources and Search Strategy

The literature search was conducted in May 2021, and involved four primary databases: Web of Science, Ovid MEDLINE, Scopus, and EBSCOhost. The keywords used to search for the related articles are listed in Supplementary Materials Table S1. There were 1342 potentially relevant records identified from the four databases. A total of 189 duplicate records were found and removed. Using automation tools, 487 records were excluded based on year, publication type, and language, leaving 666 records for title screening. The records were exported from the databases and arranged for screening in an Excel sheet.

### 2.3. Inclusion and Exclusion Criteria

The inclusion criteria were: (1) publication within the 15-year span from 2005 to 2020; (2) full original article in a journal; (3) published in English; (4) related to meteorological factors and the morbidity rates of dengue, malaria, cholera, and leptospirosis. The exclusion criteria were: (1) non-original articles such as conference proceedings, reports, systematic reviews, and meta-analyses; (2) related to vector or agent distribution in which dengue and malaria incidence is not a study outcome.

### 2.4. Study Selection

Two authors (MB and FSA) screened the titles and abstracts independently according to their relevance based on the review questions. We removed 585 articles during the screening; the remaining 81 articles proceeded to full-text retrieval for further assessment and eligibility. MB and FSA also assessed the article eligibility according to the inclusion and exclusion criteria. Disagreements were resolved by discussion with a third researcher (NA) to reach consensus. Forty-three articles were excluded because they focused only on spatial distribution (*n* = 43), had different study outcomes (*n* = 14) or focused only on vector distribution (*n* = 9). Subsequently, the remaining 38 articles proceeded to quality appraisal.

### 2.5. Quality Assessment

MB and NA assessed the quality of the 38 articles. The articles were evaluated using a modified scale of a checklist designed for assessing the quality of ecological studies by Dufault and Klar [24]. In this review, we chose this modified scale because we believe it is the best approach to assess ecological studies’ quality that incorporates methodological characteristics such as sample-based on the ecological unit, level of data aggregation, and analytical method [24]. The most concerning issue was using and adjusting covariates in the regression analyses to reduce the ecological bias. The quality assessment was based on 11 items, with a maximum overall score of 15 points. Supplementary Materials Table S2 presents the assessment scale adapted from Dufault and Klar [24]. The article quality was graded as low-(≤5 points), medium-(6–10 points), and high-relevance (≥11 points).

### 2.6. Data Extraction and Synthesis

**Figure 1:**
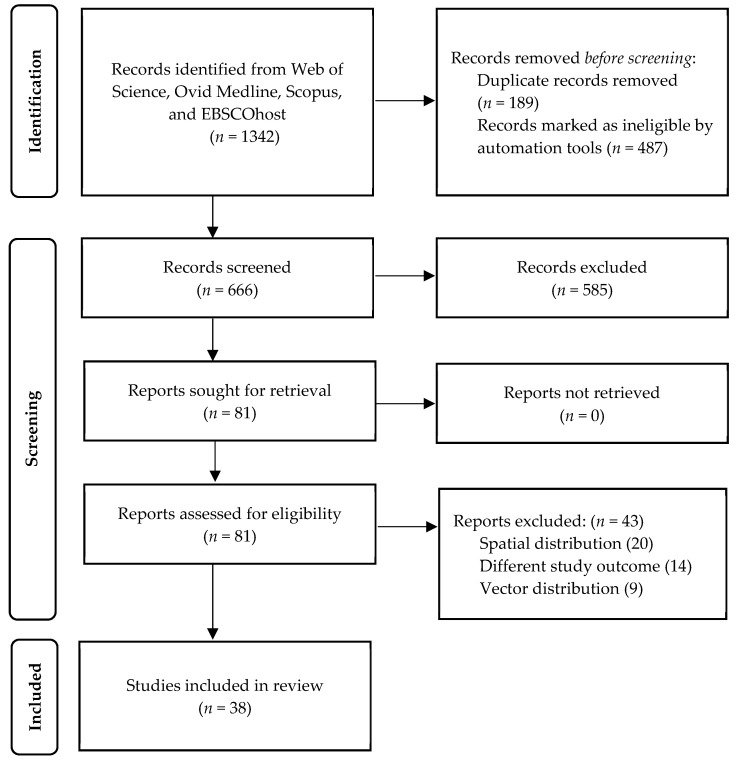
PRISMA flow diagram.

MB and NA extracted the data independently using a standardized data extraction form and organized it in a standard Microsoft Excel 2019 spreadsheet. The data and information collected included: (1) authors, (2) year of publication (3) country, (4) time frame, (5) statistical analysis and climate model, (6) findings related to meteorological factors and climate change prediction, and (7) adjustment for confounding and cross-validation. Figure 1 shows the PRISMA flow diagram. All of the studies that were chosen followed an ecological design. Due to nature of the data, which is too heterogenous in term of statistically methods, study outcomes, and settings, the quantitative synthesis and analysis was not carried out.

## 3. Results

### 3.1. Background of the Eligible Studies

A total of 38 studies were included in this systematic review. Table 1 shows a descriptive summary of the included studies. The 38 studies were conducted in Bangladesh, Brazil, China, Indonesia, India, Iran, Korea, Malaysia, Mexico, Nepal, Nigeria, Philippines, Puerto Rico, Sri Lanka, Singapore, Sudan, Taiwan, Tanzania, Thailand, and Vietnam. When categorized into World Health Organization (WHO) regions, the majority of the studies had been conducted in the Western Pacific Region (WEPRO) and South-East Asia Region (SEARO). The analyzed articles were published between 2007 and 2020. More than half of the studies (60.5%) were conducted between 2015 and 2020. The study time frame varied from ≤5 years (10.5%) to 6–10 years (52.6%). Most of the studies explored the association of meteorological factors with vector-borne diseases: 23 studies (60.5%) focused on dengue and 11 studies (29%) focused on malaria as the health outcome. Three studies and one study examined the impact of meteorological factors on cholera (7.9%) and leptospirosis (2.6%), respectively. Table 2 shows the characteristics of the included studies in terms of statistical analysis and climate model used, the association between meteorological factors and communicable disease incidence, target outcome, future prediction, adjustment for confounding factors, validation, and quality appraisal scoring.

### 3.2. Meteorological Factor Variables

The meteorological factor variables used in the included studies mainly consisted of rainfall/precipitation (34 studies), average/minimum/maximum temperature (33 studies), relative humidity (20 studies), and wind properties (three studies). Of these four meteorological factors, precipitation was most frequently associated with the selected climate-sensitive communicable diseases, followed by temperature, relative humidity, and wind properties. Of 23 studies reporting on dengue incidence, more than half reported a positive association between temperature (*n* = 17, 74%) and precipitation (*n* = 16, 70%) with dengue incidence. Furthermore, ten studies (43.5%) and three studies (13%) reported a positive association between relative humidity and wind properties, respectively, with dengue incidence. For malaria incidence, the majority of the studies reported a positive association with temperature (ten studies, 91%) and precipitation (nine studies, 81.8%). For the association between relative humidity and malaria incidence, five studies (45.5%) reported a positive association and three studies (27.3%) reported a negative association. No study reported an association between wind properties with malaria incidence. Two of three studies showed a positive association between temperature and precipitation with cholera cases. Additionally, Magny et al. [59] reported a positive association between chlorophyll A anomaly with cholera cases, while Reyburn et al. [60] reported a negative association. Lastly, Dhewantara et al. reported a positive association between temperature and precipitation with leptospirosis [61]. Table 3 summarizes the meteorological factors associated with the selected climate-sensitive communicable diseases. The other covariates included in the studies were duration of sunshine, El Niño-Southern Oscillation (ENSO), Indian Ocean Dipole (IOD), sea surface height (SSH), sea surface temperature (SST), sea level pressure, ocean chlorophyll concentration (OCC), and average river level. Some of the studies also included covariates related to landscape, such as normalized difference vegetation index (NDVI) and modified normalized difference water index (MNDWI), as well as covariates related to socio-economics, such as piped-water access, human migration, and population growth.

### 3.3. Projection of Climate-Sensitive Communicable Diseases

For the projection of dengue incidence in the event of climate change, Colon–Gonzales et al. reported that, under three climate scenarios (A1B, A2, B1), the mean annual dengue incidence across Mexico would increase around 12–18% by 2030, 22–31% by 2050, and 33–42% by 2080 [30]. A similar study conducted in Dhaka, Bangladesh, reported that dengue incidence would increase by 1.5 times if ambient temperatures increased by 1 °C in 2100 relative to 2010. If the temperature increases by 2 °C, the incidence of dengue would increase by seven times, and the worst-case scenario of a 3.3 °C rise would increase dengue incidence in Dhaka by 43 times in 2100 relative to 2010 [27]. In Guangzhou, China, Li et al. reported that under climate scenario Representative Concentration Pathway (RCP) 2.6, the overall incidence of dengue fever would be low, as would the occurrence of high numbers of cases. However, the overall incidence and the occurrence of high numbers of cases would increase under climate scenario RCP8.5 [37].

For malaria projection, Kwak et al. reported a gradually increased trend of malaria in Korea during a simulation using the RCP4.5 climate change scenario and the CNCM3 climate model. The maximum occurrence shifted from August (during 2010–2011) to July (using simulation data of 2011–2100). In the future, malaria occurrence would continually increase between April and July (before the rainy season in the summer) compared to between June and August in 2010–2011 [52]. Asadgol et al. reported that, under RCP8.5, the trend of cholera cases in Iran would increase by 2050. For the next 30 years, the seasonal pattern of cholera will change and the highest cases will be observed during spring and summer. The average monthly cholera cases will be highest in August if compared to the baseline data [11].

### 3.4. Critical Appraisal of the Studies

The studies were appraised using a modified scale of a checklist for assessing ecological research quality [24]. None of the studies were scored as low relevance: 20 studies (52.6%) and 18 studies (47.4%) were scored as high and medium relevance on the assessment scale. For level of data aggregation, 8 (21%) studies were conducted at the national level, 11 (29%) conducted at regional or state level, and the remaining 19 (50%) involved province, county, district, or city as population unit. Only two (5.3%) studies used basic Spearman’s rank and Pearson correlation as analytical methods. The rest of the studies applied advance statistical analysis such as Linear regression or Poisson regression, Autoregressive integrated moving average (ARIMA), and Multilevel Distributed Lag Non-linear Model (MDLNM). A total of 27 (71%) studies were conducted with a proper adjustment for covariates as suggested for ecological studies. For “quality of reporting”, only eight (38%) studies include a statement of ecological study design. However, 13 (34.2%) studies explicitly justified study design, and most (97.4%) of the studies discussed the risk of ecological bias. The justification of study design allows readers to understand the rationale of choosing the design and apply the ecologic analysis. The risk of ecological bias needs to be explained clearly so that the authors sufficiently caution the readers in interpreting the results, representing the aggregated level. Table 4 presents the scores of modified scale for each quality assessment item adapted from Dufault and Klar [24].

## 4. Discussion

The aim of the present systematic review was to summarize key findings related to the relationship between meteorological factors and the occurrence of dengue, malaria, cholera, and leptospirosis, and to review recent communicable disease projection in the event of global warming. The present systematic review of 38 publications demonstrates that dengue, malaria, cholera, and leptospirosis transmission can be influenced by meteorological variables such as temperature, precipitation, relative humidity, and wind properties. The actions of these climate variables in influencing the transmission of communicable diseases are rarely independent. The combinations of a few climatic variables appear to be related to climatological niches for optimal disease transmission [62]. Although the effects of climate change have been observed worldwide, the extent and patterns of the effect differ based on the country’s location and socio-economic conditions [6].

The present review shows that both precipitation and temperature are the most important meteorological factors for climate-sensitive communicable diseases, especially dengue and malaria. Several studies of different ecological units varying from city [37,39,45], province [31], regional [26,29,40], to national [41,47] levels have demonstrated a positive association between temperature and precipitation with dengue incidence. This result is not limited to studies conducted in countries with tropical climates, but also includes studies conducted in Guangzhou, which has a subtropical monsoon climate [37,45]. However, meteorological factors do not directly influence the incidence of dengue. Instead, meteorological variables such as temperatures, rainfall, and relative humidity have a direct impact on the larval development period, larval and adult mosquito survival, and the duration of the gonotrophic cycle of the primary dengue vector, and affect the general activity of the dengue vector, including host-seeking and blood meal intake [63].

### 4.1. Relationship between Metreological Factors and Dengue

The ambient temperature alters the vector population dynamic by affecting the development of immature stages and reproductive behavior [64]. The ideal temperature for Aedes aegypti development is between 22 and 32 ℃, while that for the A. aegypti adult lifespan and fecundity is between 22 and 28 ℃ [65]. Increasing the temperature will shorten the egg-laying time of A. aegypti, thereby increasing egg quantity [66]. Moreover, higher temperatures are a favorable survival range for the vector and reduce the extrinsic incubation periods of the dengue virus. This will result in higher rates of viral transmission that can lead to increased dengue incidence [67]. On the other hand, three studies have demonstrated a negative association between temperature and dengue [33,35] and dengue haemorrhagic fever (DHF) [43]. According to Duarte et al. [33], the monthly incidence of dengue will decrease by 32% with every 1 °C increment in the monthly average maximum temperature. This effect is particularly for increasing the maximum temperature to >32 °C, which is higher than the temperatures considered optimum for the vector. These temperatures help hasten the evaporation and drying of wastewater distributed around the city that would otherwise create mosquito breeding grounds.

Rainfall has also been highly associated with dengue fever incidence [26,29,32,35,37,38,39,40,41,42,43,45,46,47]. Rainfall provides abundant outdoor breeding sites for A. aegypti, for example, containers such as drums, discarded tires, and leaf axils that are naturally filled with rainwater. However, rainfall has also been negatively associated with dengue incidence in French Guiana [25], Singapore [34], Sulawesi, Indonesia [44], and Rio Branco, Brazil [33]. According to the literature [68,69], heavy rainfall may reduce mosquito density because most mosquito eggs and larvae are carried away from breeding sites. This theory might explain why dengue in the abovementioned areas is reduced as rainfall increases. Relative humidity has also been associated with dengue incidence [27,28,36,37,38,39,41,43,45,46]. Relative humidity affects all stages of the mosquito life cycle, the survival rate of the mosquito, the number of blood meals, and eventually its capacity to become infected and transmit dengue [70].

Wind speed and direction are also important climatic factors. According to Chumpu et al. the best-fit model of Phayao province, Thailand, which incorporated wind direction and wind power, showed the highest dengue occurrences at wind speeds of 5–6 knots. This indicates that wind power is crucial for the spreading of dengue by mosquitoes. A higher wind power may affect dengue fever cases. More wind power on the sea surface results in a greater evaporation zone. Adult mosquitos may be able to survive longer and spread dengue as a result of the increased humidity. For mountainous areas, the most significant meteorological factors are wind direction variables [31]. On the other hand, two studies conducted in the central region in Malaysia [29] and in Guangzhou [45] found that strong wind may suppress mosquito host-seeking activity and consequently reduce dengue transmission risk. However, only three studies included utilizing the wind properties as the independent variables. Future studies are recommended to explore wind properties as a possible meteorological factor related to dengue to overcome this limitation.

### 4.2. Relationship between Metreological Factors and Malaria

Additionally, the majority of the 11 studies on malaria included in the present review reported a positive association between temperature and precipitation with malaria incidence [48,49,50,51,52,55,56,58]. According to the data, increases in temperature, humidity, and rainfall facilitate the proliferation of mosquito populations at high altitudes. This expands the geographical distribution of malaria, allowing it to spread to new areas where mosquito populations previously did not exist. Furthermore, rising temperatures at lower altitudes, where mosquitoes and malaria are already endemic, alter the development cycle of the parasite that causes the Anopheles mosquito to transmit the disease, allowing it to develop malaria faster and therefore raising transmission rates [9]. Other than climatic factors, factors such as human migration, population growth, and deforestation are associated with malaria transmission. The relative contribution of these factors may vary between countries and regions. Furthermore, malaria transmission can be exacerbated by human behavior, such as actively storing water in open containers, routine outdoor socializing during peak hours of Anopheles biting time (dawn and dusk), and other activities such as farming and fishing, which may increase the risk of exposure to mosquitoes and malaria infection.

### 4.3. Relationship between Metreological Factors with Cholera and Leptospirosis

There is substantial evidence [11] that cholera infection is linked to meteorological factors, such as low precipitation and high temperatures during the summer months, which might facilitate bacterial reproduction and increase cholera incidence. However, in Zanzibar, East Africa, Reyburn et al. [60] reported that a 1 °C increase (at a four-month lag) would lead to two-fold increased cholera cases. Meanwhile, an increase of 200 mm rainfall (at a two-month lag) might increase cholera cases by 1.6-fold. Interestingly, a study in Matlab, Bangladesh, demonstrated a statistically significant one-month lag between OCC anomaly and cholera cases. Therefore, ocean and climatic trends are good predictors of cholera epidemics [59]. For leptospirosis, a study in China has shown that land surface temperature and rainfall are significantly associated with leptospirosis notification [61]. Warm temperature aids leptospire survival in the environment [71,72]. Hot weather encourages some activities, such as people and animals swimming in the same pool of water, e.g., rivers. Besides, high humidity is a favorable condition for leptospire survival [73]; however, Dhewantara et al. [61] did not include relative humidity as one of the covariates.

### 4.4. Projection of Selected Climate-Senstive Communicable Disease

The association between human activities and climate change has drawn increasing attention in recent years. It has been confirmed that human activity has a significant impact on present global warming. The rising emission of greenhouse gases has led to global warming and climate change, which have had various impacts, including on health, particularly toward communicable diseases [74]. Projection of the geographic distribution of A. aegypti and A. albopictus has revealed that the abundance of mosquitoes will increase by the 2030s and beyond compared to the present, suggesting that more individuals will be at risk of dengue fever [7,75,76]. A few studies have projected that exposure to the Aedes mosquito and the Aedes-transmitted virus would increase with 1.5/2.0 °C global warming [76,77]. For example, most of the tropics are now ideal for virus transmission year-round for both Aedes aegypti and Aedes albopictus, with suitability decreasing along latitudinal gradients. However, projected warming temperatures in 2050 will substantially increase the potential for year-round transmission in the tropics, even into previously protected high-elevation locations. In addition, many temperate regions are presently devoid of significant Aedes vectors. However, in 2050, the risk of Ae. albopictus transmission is projected to increase significantly in temperate countries, particularly in high-latitude portions of Eurasia and North America [75].

Elementary modelling predicts that the rising of global temperatures would increase the rate of malaria transmission and expand its geographical distribution [78,79,80,81]. Several studies have reported that the increased malaria transmission [82] or its re-emergence [83] is associated with global warming. Khormi and Kumar projected that the southern regions of China might become susceptible to malaria mosquito infection in the future, in which suitability is expected to increase. Anopheles would be able to survive in large regions of southern China that are now unsuitable or marginal [82]. Due to the high population density in these highly suitable areas, the number of individuals exposed to the Anopheles mosquito and hence to malaria is considerably increased. The predictive results indicate that modelling aids understanding of the disease transmission mechanism and assists in communicable disease intervention and control programs. However, the fundamental challenge for predicting climate-sensitive communicable disease transmission is how future climates can be best modelled at regional and/or local level. In other words, how can the results of global climate models (GCM) be suitably downscaled to a regional and/or local level? The Intergovernmental Panel on Climate Change (IPCC) Fifth Assessment Report (AR5) describes different climate futures, all of which are considered possible depending on the volume of greenhouse gases emitted in the years to come. These scenarios are categorized into four classes: RCP2.6, RCP4.5, RCP6, and RCP8.5, labelled after a possible range of radiative forcing values in 2100. These scenarios can be used to project future climates based on GCM [84].

### 4.5. Strenghts and Limitations

This review highlights the current public health issues on climate-sensitive communicable disease. All papers included in this review had undergone systematic critical appraisal using an adapted appraisal tool suitable for ecological study design, as described by Dufault and Klar [24]. Thus, we used and adapted the structured assessment scale reported in other ecological review studies [85,86]. None of the papers included in this review were low relevance, as based on the quality appraisal score. However, we recommend caution when estimating the relationships between climate variables and dengue in the following aspects: use of time lags, analysis of extreme climatic events, differences between seasonal and long-term trends, nonlinear effects and threshold effects in the associations. In addition, there should be more emphasis on data quality and the use of information for decision making.

One limitation of this review is the included articles related to leptospirosis and cholera are limited. Therefore, caution is advised for interpretation when utilizing findings related to leptospirosis and cholera. Another limitation is that very few studies use the IPCC standardized climate change scenarios to predict future dengue, malaria, and cholera incidence. Besides, the exclusion criteria of non-English language articles could be one of the limitations of this review. Nearly half of the included studies were from WEPRO, which majority comprises of non-English speaking countries. Therefore, this review might miss the wealth of related literature in particular published in Chinese. Due to the “English-language bias”, this review could have bias estimates of effect, therefore reduce its generalizability. However, including studies published in non-English language may pose additional resources with respect to cost, time, and non-English language proficiency.

One of the strengths of using the IPCC AR5 climate model is its ability to predict climates over a longer time or glacial year. The disadvantage is that it only considers the natural Earth systems and not the interaction between humans and nature. Furthermore, most of the individual studies assumed that human hosts are immobile. However, mass migration may contribute to dengue and malaria infection dynamics, especially at scales that exceed the limits of mosquito dispersal [50].

We recommend that for future research to better understand dengue, malaria, cholera, and leptospirosis ecology to be directed at predicting the climate–biological relationships on disease transmission. Uncertainties due to confounding effects of urbanization, population growth, and human migration are required to develop scenarios based on future projections of population growth and socio-economic development, including human behavior. Future projection of climate-sensitive communicable diseases is greatly essential to aid planning and mitigation strategies by stakeholders, hence the need for scientific consensus on data potentially used in modelling.

## 5. Conclusions

This review provides robust evidence of an association between meteorological factors and the incidence of climate-sensitive communicable diseases, i.e., dengue, malaria, cholera, and leptospirosis. Precipitation and temperature are important meteorological factors that influence the incidence of climate-sensitive communicable diseases. Future studies need to consider more determinants affecting precipitation and temperature fluctuations for better simulation and prediction of the incidence of climate-sensitive communicable diseases. In addition to future forecasts, accounting for alternative climate factor variables, considering climate change scenarios and other non-climatic drivers such as the presence/absence of dengue and malaria vectors, human migration, population growth, and socio-economics as crucial factors triggering communicable disease transmission would be beneficial. This would strengthen projection realism and act as a platform for academic and policymaker consensus on provisions to mitigate future climate-sensitive communicable diseases incidence.

## Figures and Tables

**Table 1:** Descriptive summary of included studies (*n* = 38).

Characteristic	Frequencies
WHO geographical region	
African Region (AFRO)	4 (10.5%)
Region of the Americas (PAHO)	4 (10.5%)
South-East Asia Region (SEARO)	12 (32%)
Eastern Mediterranean Region (EMRO)	2 (5%)
Western Pacific Region (WEPRO)	16 (42%)
Publication year	
2005–2009	3 (8%)
2010–2014	12 (31.5%)
2015–2020	23 (60.5)
Time frame	
≤5 years	4 (10.6%)
6–10 years	20 (52.6%)
11–15 years	6 (15.8%)
16–20 years	5 (13%)
≥21	3 (8%)
Health outcome (communicable disease)	
Dengue	23 (60.5%)
Malaria	11 (29%)
Cholera	3 (7.9%)
Leptospirosis	1 (2.6%)

**Table 2:** Characteristics of all the included studies.

Author, Year	Study Region	Time Frame (Years)	Statistical Analysis & Climate Model	Association between Meteorological Factors with Communicable Disease Incidence				Target Outcome	Future Prediction	Adjustment for Confounding Factors	Cross Validation	Quality Score
				Temperature	Relative Humidity	Precipitation	Other Factors					
Adde et al., 2016 [25]	French Guiana, region of France	1991–2013 (22 years)	Time-lagged Spearman’s correlations, composite analysis, logistic binomial regression model	No significant correlation	No significant correlation	−VE	Not studied	DF outbreak	French Guiana would likely experience an outbreak (probability of 0.92) in 2016.	No adjustments	Validated	10
Arcari et al., 2007 [26]	Indonesia	1992–2001 (10 years)	Pearson correlation. Stepwise multiple regression analyses.	+VE	No significant correlation	+VE	Not studied	DI/DHF I	Not studied	No adjustments	Not mention	12
Banu et al., 2014 [27]	Bangladesh (Dhaka)	2000–2010 (11 years)	Spearman’s correlation. Poisson time series model combined with DLM	+VE	+VE	No significant correlation	Not studied	DI	If 1 °C T increase in 2100, an increase of 583 DF cases. If 2 °C T increase, increase of 2782 DF cases. If T increase by 3.3 °C, increase of 16,030 cases.	Adjusted	Validated	12
Banu et al., 2015 [28]	Bangladesh	2000–2012 (12 years)	Wavelet coherence analysis, DLMN, Poisson time series model	+VE (Nino3.4 & DMI)	+VE	No significant correlation	Not studied	DI	Not studied	Adjusted for temperature, rainfall, DMI and Nino 3.4	Validated	13
Cheong et al., 2013 [29]	Malaysia (Selangor, KL, and Putrajaya)	2008–2010 (2 years)	Correlation analyses, Poisson GAM, DLNM	+VE	No significant correlation	+VE	−VE	DI	Not studied	Seasonal trends	Validated	11
Colo’ n–Gonza’ lez et al., 2013 [30]	Mexico	1985–2007 (22 years)	GAM Projected changes for the years 2030, 2050, and 2080 under three climate change scenarios (A1B, A2, and B1).	+VE	Not studied	No association	Not studied	DI	Mean annual DI may increase by about 12–18% by 2030, 22–31% by 2050, and 33–42% by 2080.	No adjustments	Validated	10
Chumpu et al., 2019 [31]	Thailand	2001–2014 (15 years)	Generalized linear models (Poisson regression, negative binomial regression, quasi likelihood Regression) ARIMA and SARIMA	+VE	No significant correlation	+/−VEdepending on province	+VE	DI	Not studied	Adjusted	Validated	14
Chuang et al., 2017 [32]	Taiwan (South western)	1998–2015 (8 years)	DLNM, Wavelet analysis	+VE	Not studied	+VE	Not studied	DI	Not studied	Adjusted	Validated	11
Duarte et al., 2018 [33]	Rio Branco, Brazil	2001–2012 (12 years)	Generalized autoregressive moving average models with negative binomial distribution	−VE	−VE	−VE	Not studied	DI	Not studied	Adjusted	Not mention	13
Hii et al., 2009 [34]	Singapore	2000–2007 (8 years)	Time series Poisson regression model	+VE	Not studied	−VE	Not studied	DI	Not studied	No adjustments	Validated	10
Iguchi et al., 2018 [35]	Davao Region, Philippines	2011–2015 (5 years)	A quasi-Poisson time series model coupled with DLNM.	−VE	Not studied	+VE	Not studied	DI	Not studied	Adjusted	Not mention	11
Jiang et al., 2017 [36]	San Juan, Puerto Rico	1990–2013 (23 years)	K-nearest neighbor (KNN) regression	+VE	+VE	No significant correlation	Not studied	DI	Regression prediction Error (RMSE) is 6.88 person/week.	No adjustments	Validated	9
Li et al., 2017 [37]	Guangzhou, China	1998–2014 (17 years)	Spearman rank coefficient and Pearson correlation coefficient. Generalized additive model (GAM)	+VE	+VE	+VE	+VE	DI	Under RCP 2.6, overall incidence of DF is low Under RCP 8.5, both the overall incidence and occurrence of high numbers of cases increase.	No adjustments	Validated	8
Minn An & Rocklöv 2014 [38]	Vietnam (Hanoi)	2002–2010 (9 years)	Stepwise multivariate linear regression analysis	+VE	+VE	+VE	Not studied	DI	Not studied	Bonferroni corrections	Validated	8
Noureldin & Shaffer 2019 [39]	Sudan (Port Sudan)	2008–2013 (6 years)	Wilcoxon rank sum test and multiple linear regression	+VE	+VE	+VE	Not studied	DI	No adjustments	Not mention	Not studied	9
Pham et al., 2020 [40]	Vietnam (Mekong Delta region)	2000–2016 (17 years)	ARIMA	+VE	Not studied	+VE	Not studied	DI	DF incidence mostly in rainy seasons	No adjustments	Validated	14
Sirisena et al., 2017 [41]	Sri Lanka	2009–2014 (6 years)	Spearman’s correlation	+VE	+VE	+VE	Not studied	DI	Not studied	No adjustments	Not mention	12
Sharmin et al., 2015 [42]	Dhaka, Bangladesh	2000–2009 (10 years)	Spearman’s rank correlation test. Negative binomial generalized linear model.	+VE	No significant correlation	+VE	Not studied	DI	Not studied	Adjusted	Not mention	8
Tang et al., 2020 [43]	Indonesia (Surabaya, East Java)	2009–2017 (9 years)	One-Sample Kolmogorov–Smirnov Test, Spearman non-parametric correlation test.	−VE	+VE	+VE	Not studied	DHF I	Not studied	No adjustments	Not mention	8
Tosepu et al., 2017 [44]	Sulawesi, Indonesia	2010–2015 (6 years)	Spearman and time-series Poisson multivariate regression. GEE with a Poisson distribution	+VE	−VE	−VE	Not studied	DHF I	Not studied	Adjusted	Not mention	10
Xiang et al., 2017 [45]	Guangdong, China	2005– 2014 (10 years)	DLNM, GEE with negative binominal distribution.	+VE	+VE	+VE	−VE	DI	Not studied	Adjusted	Not mention	11
Xuan et al., 2014 [46]	Vietnam	2008–2012. (5 years)	Poisson regression model	No association	+VE	+VE	Not studied	DI	Not studied	Adjusted	Not mention	10
Xu et al., 2014 [47]	Singapore	2001–2009 (9 years)	Spearman rank correlation analysis. Poisson regression combined with DLNM	+VE	Not significant	+VE	Not significant	DI	Not studied	Adjusted	Not mention	12
Akinbobola & Omotosho 2011 [48]	Nigeria (Akure city)	2001–2007 (7 years)	ARIMA	+VE	+VE	+VE	Not studied	MI	Not studied	Not adjusted	Not mention	9
Bhandari et al., 2013 [49]	Nepal (Jhapa district)	1999–2008 (10 years)	ARIMA	+VE	No significant correlation	+VE	Not studied	MI	Not studied	Not adjusted	Not mention	8
Gao et al., 2012 [50]	China (Anhui province)	1990–2009 (20 years)	Spearman correlations. Polynomial distributed lag (PDL) time-series regression	+VE	+VE	+VE	+VE with El Niñ o/Southern Oscillation	MI	Not studied	Adjusted	Validated	12
Jones et al., 2007 [51]	North-west Tanzania	1990–1999 (10 years)	Multiple linear regression analysis	+VE	No significant correlation	+VE	Not studied	MI	Not studied	Adjusted	Validated	9
Kwak et al., 2014 [52]	Korea	2001–2011 (11 years)	Spectral analysis. Brock–Dechert–Scheinkman (BDS) Statistic. Nonlinear Regression Analysis. PCA-Regression Analysis	+VE	+VE	+VE	Not studied	MI	Under RCP 4.5, malaria occurrence trend will gradually increase. Malaria occurrence will increase before the rainy season in summer (April and July).	Adjusted	Not mention	12
Ostovar et al., 2016 [53]	Iran	2003–2009 (7 years)	ARIMA models with Transfer Function.	+VE	−VE	No significant correlation	Not studied	MI	Not studied	Adjusted	Not mention	9
Rejeki et al., 2018 [54]	Indonesia	2005–2014 (10 years)	Poisson model, quasi–Poisson model, and negative binomial model	No significant association.	−VE	+VE	Not studied	MI	Not studied	Not adjusted	Not mention	11
Sehgal et al., 2020 [55]	India (Andhra Pradesh)	2014–2016 (3 years)	GLM with Poisson distribution. Quasi-Poisson method with GAM	+VE	−VE	+VE	Not studied	MI	Not studied	Adjusted	Not mention	10
Wardrop et al., 2013 [56]	Yunnan Province, China	1991–2006 (16 years)	Poisson regression with distributed lag non-linear	+VE	Not studied	+VE	Not studied	MI	Not studied	Adjusted	Not mention	10
Xiang et al., 2018 [57]	Anhui, Henan, and Yunnan Provinces China	2005–2012 (8 years)	Generalized estimating equation models with negative binominal distribution.	+VE	+VE	−VE	Not studied	MI	Not studied	Adjusted	Not mention	13
Zhao et al., 2014 [58]	South-west China	2004–2009 (6 years)	Multilevel Distributed Lag Non-linear Model(MDLNM)	+VE	+VE	+VE	Not studied	MI	Not studied	Adjusted	Not mention	13
Asadgol et al., 2019 [11]	Iran	1998–2016 (19 years)	Artificial Neural Networks (ANNs)	+VE	Not studied	−VE	Not studied	Cholera cases	Under RCP8.5, the cholera trend will increase by the year 2050.	Adjusted	Validated	11
Magny et al., 2008 [59]	Kolkata, India, and Matlab, Bangladesh	1998–2006 (9 years)	GLM with a Poisson distribution and a log link	No significant correlation	Not studied	+VE	+VE CHL anomaly	Cholera epidemic	Not studied	Adjusted	Validated	12
Reyburn et al., 2011 [60]	Zanzibar, East Africa	1997–2006 (10 years)	SARIMA	+VE	Not studied	+VE	No significant correlation with CHLano	Cholera cases	Not studied	Adjusted	Validated	10
Dhewantara et al., 2019 [61]	China (Megla and Yunan county)	2006–2016 (11 years)	Time series cross-correlation analysis	+VE	Not studied	+VE	Not studied	Leptospirosis notification	Not studied	Adjusted	Validated	12

ARIMA: Autoregressive integrated moving average, CHLano: Chlorophyll concentration anomaly DLNM: Distributed lag non-linear models, DI: Dengue incidence, DHF: Dengue Hemorrhagic Fever, GAM: generalized additive models, GEE: Generalized estimating equations, GLM: Generalized linear model, MI: Malaria incidence.

**Table 3:** Summaries of mereological factors associated with selected climate-sensitive communicable disease.

	Temperature	Relative Humidity	Precipitation	Wind Properties
	*n* (%)	*n* (%)	*n* (%)	*n* (%)
Dengue (23 studies)				
Positive association	17 (74)	10 (43.5)	16 (70)	2 (8.7)
Negative association	3 (13)	2 (8.7)	3 (13)	2 (8.7)
Positive and negative association			1 (4.3)	
Total	20	12	20	4
Malaria (11 studies)				
Positive association	10 (91)	5 (45.5)	9 (81.8)	0
Negative association	0	3 (27.3)	1 (9.1)	0
Total	10	8	10	0
Cholera (3 studies)				
Positive association	2 (66.7)	0	2 (66.7)	0
Negative association	0	0	1 (33.3)	0
Total	2	0	3	0
Leptospirosis (1 study)				
Positive association	1 (100)	0	1 (100)	0
Negative association	0	0	0	0
Total	1	0	1	0
Grand total	33	20	34	3

**Table 4:** Critical appraisal of selected studies. Modified scale from Dufault and Klar [24].

Author (Year)	Study Design and Focus				Statistical Methodology				Quality of Reporting			Score
	Sample Size	Level of Data Aggregation	Level of Inference	Pre-Specification of Ecologic Units	Analytic Methodology	Validity of Statistical Inferences	Use of Covariates	Proper Adjustment for Covariates	Statement of Study Design	Justification of Study Design	Discussion of Cross-Level Bias and Limitations	Points
Adde et al., 2016 [25]	2	2	1	1	2	0	1	0	0	0	1	10
Arcari et al., 2007 [26]	2	2	1	1	2	1	1	1	0	0	1	12
Banu et al., 2014 [27]	2	3	1	1	2	0	1	1	0	0	1	12
Banu et al., 2015 [28]	2	3	1	1	2	0	1	1	0	1	1	13
Cheong et al., 2013 [29]	1	2	1	1	2	1	1	1	0	0	1	11
Colo’n–Gonza’ lez et al., 2013 [30]	2	2	1	1	2	0	1	0	0	0	1	10
Chumpu et al., 2019 [31]	2	3	1	1	2	1	1	1	0	1	1	14
Chuang et al., 2017 [32]	2	1	1	1	2	1	1	1	0	0	1	11
Duarte et al., 2018 [33]	2	1	1	1	2	1	1	1	1	1	1	13
Hii et al., 2009 [34]	2	3	1	0	2	1	0	0	0	0	1	10
Iguchi et al., 2018 [35]	2	2	1	0	2	1	1	1	0	0	1	11
Jiang et al., 2017 [36]	2	2	1	0	2	1	1	0	0	0	0	9
Li et al., 2017 [37]	1	1	1	0	2	1	1	0	0	0	1	8
Minn An & Rocklöv 2014 [38]	1	1	1	0	2	1	0	1	0	0	1	8
Noureldin & Shaffer 2019 [39]	1	1	1	0	2	1	0	0	1	1	1	9
Pham et al., 2020 [40]	2	2	1	1	2	1	1	1	1	1	1	14
Sirisena et al., 2017 [41]	2	3	1	1	2	1	0	0	0	1	1	12
Sharmin et al., 2015 [42]	1	1	1	1	1	1	0	1	0	0	1	8
Tang et al., 2020 [43]	1	1	1	1	1	1	0	0	1	0	1	8
Tosepu et al., 2017 [44]	1	1	1	1	2	1	1	1	0	0	1	10
Xiang et al., 2017 [45]	2	1	1	1	2	1	1	1	0	0	1	11
Xuan et al., 2014 [46]	1	1	1	1	2	1	1	1	0	0	1	10
Xu et al., 2014 [47]	2	3	1	1	2	1	0	1	0	0	1	12
Akinbobola & Omotosho 2011 [48]	1	1	1	1	2	1	1	0	0	0	1	9
Bhandari et al., 2013 [49]	1	1	1	1	2	1	0	0	0	0	1	8
Gao et al., 2012 [50]	2	1	1	1	2	1	1	1	0	1	1	12
Jones et al., 2007 [51]	1	2	1	1	2	0	0	1	0	0	1	9
Kwak et al., 2014 [52]	2	3	1	1	2	0	1	1	0	0	1	12
Ostovar et al., 2016 [53]	2	1	1	0	2	0	1	1	0	0	1	9
Rejeki et al., 2018 [54]	2	1	1	1	2	0	1	0	1	1	1	11
Sehgal et al., 2020 [55]	2	1	1	0	2	0	1	1	1	0	1	10
Wardrop et al., 2013 [56]	2	1	1	1	2	0	1	1	0	0	1	10
Xiang et al., 2018 [57]	2	2	1	1	2	1	1	1	0	1	1	13
Zhao et al., 2014 [58]	2	2	1	1	2	1	1	1	0	1	1	13
Asadgol et al., 2019 [11]	2	1	1	0	2	0	1	1	1	1	1	11
Magny et al., 2008 [59]	2	3	1	1	2	0	1	1	0	0	1	12
Reyburn et al., 2011 [60]	2	1	1	0	2	0	1	1	0	1	1	10
Dhewantara et al., 2019 [61]	2	2	1	0	2	0	1	1	1	1	1	12

## Data Availability

Data is contained within the article or supplementary material.

## Supplementary Materials

The following are available online at https://www.mdpi.com/article/10.3390/ijerph182111117/s1, Table S1: Keywords search used in the screening process. Table S2: Quality assessment scale adapted from Dufault and Klar.
