# Supplementary material for: The Impact of Meteorological Factors on Communicable Disease Incidence and Its Projection: A Systematic Review
Source: Int J Environ Res Public Health. 2021 Oct 22;18(21):11117. doi: 10.3390/ijerph182111117 (PMC8583681; doi:10.3390/ijerph182111117)
Supplement: Supplementary file 1 [file ijerph-18-11117-s001.zip › Supplementary material Table S1.pdf]

Table S1: Keywords search used in the screening process.

Database	Search string
Web of Science	TS=((“Global warm*” OR “Climate chang*” OR “climate warm*” OR “global heat*” OR “greenhouse effect*”) AND (relations* OR associat* OR connect*) AND (“meteorology* factor*” OR temperature* OR humid*OR precipitation* OR rainfall*) AND (“Communicable disease*” OR “infect* disease*” OR “transmit* disease*” OR “vector-borne disease*” OR “food-borne disease*” OR “water borne disease*” OR dengue OR malaria OR cholera OR leptospirosis) AND (project* OR forecast* OR predict* OR estimate*))
Ovid Medline	(Global warm* or Climate chang* or climate warm* or global heat* or greenhouse effect*) AND (relations* OR associat* OR connect*) AND (meteorology* factor* OR temperature* OR humid*OR precipitation* OR rainfall*) AND (Communicable disease* OR infect* disease* OR transmit* disease* OR vector-borne disease* OR food-borne disease* OR water borne disease* OR dengue OR malaria OR cholera OR leptospirosis) AND (project* OR forecast* OR predict* OR estimate*)
Scopus	TITLE-ABS-KEY ((“Global warm*” OR “Climate chang*” OR “climate warm*” OR “global heat*” OR “greenhouse effect*”) AND (relations* OR associat* OR connect*) AND (“meteorology* factor*” OR temperature* OR humid*OR precipitation* OR rainfall*) AND (“Communicable disease*” OR “infect* disease*” OR “transmit* disease*” OR “vector-borne disease*” OR “food-borne disease*” OR “water borne disease*” OR dengue OR malaria OR cholera OR leptospirosis) AND (project* OR forecast* OR predict* OR estimate*))
EBSCOhost	(Global warm* or Climate chang* or climate warm* or global heat* or greenhouse effect*) AND (relations* OR associat* OR connect*) AND (meteorology* factor* OR temperature* OR humid*OR precipitation* OR rainfall*) AND (Communicable disease* OR infect* disease* OR transmit* disease* OR vector-borne disease* OR food-borne disease* OR water borne disease* OR dengue OR malaria OR cholera OR leptospirosis) AND (project* OR forecast* OR predict* OR estimate*)
