# Supplementary material for: The Impact of Meteorological Factors on Communicable Disease Incidence and Its Projection: A Systematic Review
Source: Int J Environ Res Public Health. 2021 Oct 22;18(21):11117. doi: 10.3390/ijerph182111117 (PMC8583681; doi:10.3390/ijerph182111117)
Supplement: Supplementary file 1 [file ijerph-18-11117-s001.zip › Supplementary material Table S2.pdf]

Table S2. Quality assessment scale adapted from Dufault and Klar [1].

Evaluation criterion	Categories	Definition	Points (max=15)
**STUDY DESIGN (max=7)**			
Sample size	< 11% units<br>11-79% units<br>≥ 80% units	Number of ecologic units included in the analysis as proportion of the total number of units	0<br>1<br>2
Level of data aggregation	Other than below<br>Regional, State<br>National	Population to which the units refer to. "Other" may be province, county, district, city, group	1<br>2<br>3
Level of inference	Individual or unclear<br>Ecologic	The results of analysis are used to draw inferences for individuals or groups (ecologic).	0<br>1
Pre-specification of ecologic units	No<br>Yes	Ecologic units are selected to suit the hypothesis? (as opposed to selection motivated by convenience or necessity)	0<br>1
**STATISTICAL METHODOLOGY (max=5)**			
Analytic methodology	Spearman's rank correlation<br>Pearson correlation<br>Negative binomial generalized linear model<br>Linear regression analysis<br>Poisson regression<br>Autoregressive integrated moving average (ARIMA)<br>K-nearest neighbor (KNN) regression<br>Multilevel Distributed Lag Non-linear Model (MDLNM)	All statistical methods are acceptable if they are used appropriately. The score was assigned based on the complexity and flexibility of the method.<br>1 = Spearman's rank correlation, Pearson correlation, Negative binomial generalized linear model.<br>2 = Linear regression analysis, Poisson regression, Autoregressive integrated moving average (ARIMA), K-nearest neighbor (KNN) regression, Multilevel Distributed Lag Non-linear Model (MDLNM)	1<br>2
Validity of statistical inferences	No<br>Yes	The minimum number of ecological unit are 10 units per covariate	0<br>1
Use of covariates	No<br>Yes	Analysis adjusted for covariates (e.g. socio-economic, population growth)	0<br>1
Proper adjustment for covariates	No<br>Yes	Are the outcomes standardized or adjusted for certain factors before model adjustment? For standardized or adjusted outcomes, the standardized or adjusted factors should be included in the adjustment model. If standardized/adjusted outcomes are not used, this criterion is considered to have been met.	0<br>1
**QUALITY OF REPORTING (max=3)**			
Statement of study design	No<br>Yes	Key elements of study design are presented in the report	0<br>1
Justification of study design	No<br>Yes	Justification of ecological analysis, the rational and the specific objectives presented in the report	0<br>1

Discussion of cross-level bias and limitations	No<br>Yes	Readers are cautioned about the limitations of the ecological design, the ecologic fallacy and impracticality of extrapolating to a different level	0<br>1

1. Dufault B, Klar N. The Quality of Modern Cross-Sectional Ecologic Studies: A Bibliometric Review. American journal of epidemiology. 2011;174:1101-7. doi: 10.1093/aje/kwr241.
